# The Relationship between Sleep Quality and Posture: A Study on University Students

**DOI:** 10.3390/life14101244

**Published:** 2024-09-28

**Authors:** Adela Badau, Dana Badau, Sebnem Sarvan Cengiz, Ebrar Şevval Coşkun

**Affiliations:** 1 Faculty of Physical Education and Mountain Sports, Transilvania University, 500068 Brasov, Romania; 2 Faculty of Sport Sciences, Manisa Celal Bayar University, 45140 Manisa, Turkey; 3 Faculty of Sport Sciences, Istanbul Gedik University, 34876 Istanbul, Turkey

**Keywords:** body segment posture, evaluating postural alignment, head–neck, sleep quality, sports students

## Abstract

The aim of this study is to investigate body posture, physical exercises, head–neck relationship, and sleep quality among university students. A total of 96 students, with an average age of 20.86 ± 1.24 years and an average BMI of 23.41 ± 2.56, voluntarily participated in the study. The REEDCO Posture Evaluation (RPE) was used to assess the participants’ body posture scores. Head and neck measurements were taken using the Apecs-AI Posture Evaluation and Correction System^®^ (Apecs Posture Analysis Pro Plus Version 8.2.6). Sleep quality was measured using the Pittsburgh Sleep Quality Index (PSQI). Pearson correlation analysis indicated that increased caffeine consumption was associated with poorer sleep quality (r = 0.267, *p* < 0.05). Additionally, increased participation in physical activities was associated with improved sleep quality, with those engaging in sports having better sleep quality scores (r = −0.278, *p* < 0.05). As physical activity increased, REEDCO scores decreased (r = −0.423, *p* < 0.05), while scores for right head (r = 0.210, *p* < 0.05) and left head (r = 0.247, *p* < 0.05) increased. Significant negative correlations were found between REEDCO scores and right head (r = −0.296, *p* < 0.05) and left head (r = −0.463, *p* < 0.05) scores. In conclusion, due to the limited number of studies investigating head–neck relationships and sleep quality, definitive conclusions cannot be drawn; further and more comprehensive research is needed.

## 1. Introduction

Approximately one-third of human life is spent sleeping. Sleep is a vital restorative process that supports the body’s biological renewal and reduces physical fatigue [1]. However, sleep deprivation and irregular sleep patterns can lead to serious long-term health issues. Recent research indicates that students who sleep less than 9 h per night experience shrinkage in brain regions responsible for memory, intelligence, and well-being, with these changes becoming apparent within two years [2]. Therefore, sleep quality is of significant importance both for clinical practices and sleep research due to its strong association with physical and mental well-being, as well as with the prevalence of sleep disorders and the potential indication of various diseases [3]. Additionally, the complex relationships among sleep, physical activity, obesity, and posture require further in-depth research beyond current knowledge. For example, it has been observed that short sleep durations increase the risk of obesity, while long sleep durations do not have such an effect [4]. This finding is particularly surprising given that a sedentary lifestyle is generally thought to contribute to obesity. Electroencephalogram (EEG) and the evolution of sleep develop systematically from the fetus, through premature and full-term infants, to early childhood, adolescence, and adulthood, along with the maturation of the central nervous system. These ontogenetic changes can be significantly influenced by neurological, environmental, and genetic factors, as well as accompanying medical or neurological disorders. Sleep requirements change dramatically from infancy to old age [5]. The requirements for sleep duration are also related to exposure to light and dark, genetic factors, developmental age, and gender [6]. In previous studies, scientists have been investigating the effects of various exercise interventions on sleep quality [7]. The effect of exercise on sleep can be explained by the physical fatigue induced by exercise intensity, which increases deep sleep duration in the brain [8,9,10]. Moreover, aerobic exercise may prevent the flattening of the body temperature rhythm. While body temperature rises during exercise, it decreases to a level lower than pre-exercise levels after the exercise is completed. Consequently, increased fatigue post-exercise may contribute to improved sleep quality during the night [11].

University students face various challenges, including reduced parental support and increased academic workload. These challenges involve increased cognitive demands and identity formation processes, which often lead to disruptions in the sleep–wake cycle, shortened sleep duration, and delays in sleep onset. Additionally, students commonly exhibit behaviors such as sleep deprivation, poor sleep quality, and excessive daytime sleepiness [12,13]. Poor sleep quality can lead to mental issues such as depression, anxiety, stress, impaired attention, low self-esteem, and distorted body image, as well as physical health problems such as obesity and cardiovascular diseases [14,15]. This underscores the need to identify the serious health impacts on university students, a vulnerable subgroup, and to determine the risk factors necessary for managing sleep issues. This necessity arises from the negative effects on students’ cognitive abilities related to daily performance and academic success [16,17].

Studies investigating sleep quality and respiratory disorders have focused on the negative effects of supine posture on respiratory abnormalities and the relationship between sleep quality and respiratory function [18,19]. When the physiological structure of respiration is affected, the postural muscles required to meet respiratory needs are also impacted. Activities such as coughing, which utilizes the same muscles involved in respiration, can also influence posture [20]. A study investigating the effects of forward head posture on respiratory muscle strength and respiratory function tests observed a relationship between the C7 vertebral corpus angle in forward head posture and measured expiratory muscle strength and reference-adjusted expiratory muscle strength [21,22].

A review of the literature revealed a complex relationship among body posture, respiratory function, physical exercise, sleep quality, and forward head posture. In this context, the aim of the study is to investigate body posture, physical exercise, head–neck relationships, and sleep quality among university students. The hypothesis of the study started from the assumption that the quality of sleep among university students is dependent on body posture, the head–neck relationship, and the level of practicing physical activities.

## 2. Materials and Methods

### 2.1. Participants

A total of 96 participants, all university students, were included in this study. The average age (±standard deviation) of the participants was 20.86 ± 1.24 years, and the mean body mass index (BMI) was 23.41 ± 2.56. The mean front head tilt was measured at 1.43, back head tilt at 1.93, right head shift at 37.30, and left head shift at 33.65. According to the responses provided in their personal information forms, the participants’ average daily coffee consumption was 1.58, the average smoking rate was 1.72 per day, and the average participation in physical activity was 2.41 days a week. The inclusion criteria required the participants to be students, healthy, without a history of disabilities, to perform all the measurements, and to fully complete the applied questionnaire. Prior to conducting the tests, all participants were thoroughly informed about the procedures and provided verbal consent to participate voluntarily. Statistical software G*Power (v3.1.9.7) was used to calculate the sample, and it was selected for the present study effect size of 0.30, *p* value of 0.05, and power of 0.80; the analysis revealed that a total sample size of N = 64 was adequate.

### 2.2. Study Design

In this study, a cross-sectional design was used to evaluate the posture and sleep quality of university students. All evaluation methods were explained to each participant, and the collection of the results was performed physically by the authors. After performing the physical tests, the study participants were given a questionnaire each and the way to complete it was explained to them. The subjects were not subjected to any protocol prior to the tests.

### 2.3. Data Collection Tools

The following devices were used in the present study:

Anthropometric data: The following devices were used to measure the anthropometric parameters: the Seca digital thaliometer for height investigation (cm) and Tanita scale (digital) for weight assessment (kg). These two parameters were necessary for BMI calculation, and the test conditions were identical for all subjects [23].

APECS-AI Posture Evaluation and Correction System^®^ (Apecs Posture Analysis Pro Plus Version 8.2.6) is a device that can be installed on a phone and is based on precise photogrammetric algorithms for the purpose of precise posture evaluations and is used in physical therapy. The interface of the application shows a target point that turns green, thus avoiding any deviation of the camera position. After taking the photo, the application asks to cut out the body segments that are not targeted. The information provided is based on standardized digitized landmarks; a maximum of 4 images can be made depending on the variables of interest [24]. As part of the research, photographs of university students were taken in five different standing positions (“front”, “back”, “right”, “left”, and “bending position”) to analyze their body posture. Using these photographs, the students’ posterior, anterior, and lateral postures were examined. According to previous studies, the APECS mobile application is accessible, having a high reliability in posture assessment [25,26].

Reedco Posture Evaluation (RPE): The RPE is a standard method used since 1974 to evaluate the entire body posture from head to toe in the sagittal and coronal planes (Auburn, NY, USA 1974) [27]. This method assesses individuals observationally from the lateral and posterior views across 10 postural features. Lateral evaluation covers the neck, upper back, torso, abdomen, and lumbar regions in the sagittal plane, while posterior evaluation includes the head, shoulders, spine, hips, and ankles in the coronal plane. RPE scores are determined by evaluating postural alignment on a scale from “0” to “10”, where “0” indicates poor posture or significant deviation, “5” indicates fair posture or minimal–moderate deviation, and “10” indicates good posture or normal alignment. A maximum score of 100 represents good posture, while a score of 59% or lower indicates postural dysfunction [28].

Pittsburgh Sleep Quality Index (PSQI): Developed by Buysse and colleagues (1989) [29] and adapted into Turkish by Ağargün and team (1996) [30], the PSQI is a 19-item self-report scale that assesses sleep quality and disturbances over the past month. It comprises a total of 24 questions, 19 of which are individually answered, while the remaining 5 are answered by a bed partner or roommate. The 18 scored questions form seven different components: Subjective Sleep Quality, Sleep Latency, Sleep Duration, Habitual Sleep Efficiency, Sleep Disturbances, Use of Sleeping Medication, and Daytime Dysfunction. Each component is rated on a scale from 0 to 3. The sum of these component scores determines the overall score of the scale, ranging from 0 to 21. A total score greater than 5 may indicate poor sleep quality. The reliability of the questionnaire for our study, by calculating the Cronbach α index, was 0.83.

### 2.4. Statistics Analysis

The data obtained from the research were analyzed using SPSS 22.0 software aiming at the following parameters: arithmetic means (X), standard deviation (SD), variance, and kurtosis. One-way ANOVA anaylsis and Pearson correlation analysis were used to examine the relationship between variables. The statistical significance level for this study was *p* < 0.05. Cronbach’s α index was also applied to determine the reliability of the PSQI questionnaire. For the interpretation of the size effect, we considered the two limits of η^2^ = 0, highlighting that there is no relationship between the two analyzed factors, and η = 1 highlights a perfect relationship for the present study and is as follows: η^2^ = 0.01 small effect, η^2^ = 0.06 medium effect, and η^2^ = 0.14 large effect. Statical software G*Power (v3.1.9.7) was used to calculate the sample size.

### 2.5. Ethical Consideration

The procedure for conducting the study and the stages were communicated to the students, who participated voluntarily, giving their verbal consent to participate. The research was conducted in accordance with the professional and ethical standards of the Declaration of Helsinki (2013) and the agreement of the Ethics Committee of the Health Sciences no. E.765661/26.04.2023, Manisa Celal Bayar University, Faculty of Medicine.

## 3. Results

In Table 1, the arithmetic means of the main parameters recorded in the applied tests are presented, and when interpreting the results, we referred to the minimum and maximum values.

A one-way ANOVA analysis was conducted to understand the impact of caffeine consumption on sleep quality. The analysis revealed significant differences in sleep quality based on caffeine consumption (f(3.92) = 4.157; *p* < 0.05; η^2^ = 0.119). According to the results, participants who consumed 6 or more units of caffeine per day had significantly poorer sleep quality compared to other participants.

A one-way ANOVA analysis was conducted to understand the impact of participation in physical activity on sleep quality. The analysis revealed significant differences in sleep quality based on participation in physical activity (f(3.92) = 3.438; *p* < 0.05; η^2^ = 0.101). According to the results, participants who did not participate in physical activity had significantly poorer sleep quality compared to those who engaged in physical activity 3–5 days per week (Table 2).

A one-way ANOVA analysis was conducted to determine whether there were differences in REEDCO scores based on participation in physical activity. The analysis revealed significant differences in REEDCO scores according to physical activity participation (f(3.92) = 7.698; *p* < 0.01; η^2^ = 0.201). According to the results, participants who engaged in physical activity 7 days a week had significantly lower REEDCO scores compared to other participants (Table 3).

According to the Pearson correlation analysis, an increase in caffeine consumption among participants was associated with an increase in poor sleep quality (r = 0.267, *p* < 0.05). Additionally, increased participation in physical activities was associated with a decrease in poor sleep quality, indicating that those who engaged in sports had better sleep quality scores (r = −0.278, *p* < 0.05). Furthermore, as physical activity increased, REEDCO scores decreased (r = −0.423, *p* < 0.05), while right head (r = 0.210, *p* < 0.05) and left head (r = 0.247, *p* < 0.05) scores increased. There were significant negative correlations between REEDCO scores and both right head (r = −0.296, *p* < 0.05) and left head (r = −0.463, *p* < 0.05) scores. Finally, participants who engaged in more sports activities tended to smoke more (r = 0.217, *p* < 0.05) and an increase in age was associated with a higher caffeine intake (r = 0.269, *p* < 0.05) (Table 4).

## 4. Discussion

The literature contains numerous studies demonstrating that reduced sleep duration can lead to postural control disorders. It is emphasized that for postural control disorders to manifest, at least two of the three sensory systems (somatosensory, visual, and vestibular) involved in postural control must be impaired, with one of these systems necessarily being the vestibular system. Sleep problems increase the demands on the sensory systems, and these demands are met through a top-down regulation strategy by the central nervous system [31,32]. However, in our study, no significant relationship was found between sleep quality and postural scores (REEDCO). As noted by Fabbri et al. (2006) [33], visual disturbances and changes in sensory integration resulting from sleep problems can lead to postural control problems. However, similar to our study, Iyigun et al. (2017) [14] found no significant relationship between New York postural scores and the Pittsburgh Sleep Quality Index (PSQI) in their study on university students. In another study, participants subjected to 24 to 36 h of sleep deprivation did not show a deterioration in postural performance as expected; on the contrary, some signs of improvement were reported [34,35].

A study investigating the effects of different times of day and sleep deprivation on postural control found that the impact of sleep deprivation on postural control varied depending on the time of day. At 6:00 a.m., sleep deprivation had no effect on postural control. However, at 10:00 a.m. and 2:00 p.m., sleep deprivation caused significant increases in COP surface area and LFS ratio. At 6:00 p.m., while the LFS ratio increased, the COP surface area returned to its 6:00 a.m. level [36]. In our study, no specific time protocol was applied in the postural assessments. It was reported that reduced sleep duration not only failed to lead to a decrease in postural control but also is a condition that is related to the time of day, body temperature, and daily fluctuations in postural control, with these fluctuations being primarily connected to the vestibular system [37,38,39].

In our study, significant negative correlations were found between REEDCO scores and right head (r = −0.296; *p* < 0.05) and left head (r = −0.463; *p* < 0.05) scores. However, no significant relationship was found between right head and left head scores and PSQI. Upon examining the findings, the significant negative correlations between body posture and head–neck scores may be due to better body posture. Nevertheless, it is suggested that better respiratory function, proportionate to better head–neck scores, does not have an impact on sleep quality. Further research is needed on this topic.

Current data indicate that the relationship between exercise frequency and sleep quality is not statistically significant (f(7, 88) = 1.890, *p* = 0.081). However, individuals who engage in regular exercise tend to have lower Pittsburgh Sleep Quality Index (PSQI) scores, suggesting better sleep quality. A study by Alhusami et al. (2024) [40] on health sciences students found that those who engage in regular physical activity have higher sleep quality. Similarly, Merellano-Navarro et al. (2022) [41] found that high levels of physical activity and being male positively influenced overall PSQI scores among physical education students. Xu et al. (2023) [7] investigated the effects of physical exercise on sleep quality among university students and found that those who exercised had lower PSQI scores (r = −0.159, *p* < 0.001) and identified a significant relationship between physical exercise and sleep quality (r = −0.159, *p* < 0.001).

The relationship between exercise frequency and sleep quality is an important issue for university students. Review studies have shown that university students generally have low levels of physical activity and that one in three students is overweight or obese. Additionally, only one-fifth of students engage in sufficient physical activity for health purposes. Notably, among female students, lower physical activity scores are associated with increased depression scores, and lower physical activity scores are related to higher anxiety scores. Research also indicates that students’ sitting times are higher than their walking times and moderate physical activity scores, but lower than their vigorous physical activity scores. Furthermore, there is a positive relationship between physical activity level and academic success and a negative relationship with body mass index [42]. According to the Pearson correlation analysis in our study, as participants’ caffeine consumption increased, there were declines in sleep quality (r = 0.267, *p* < 0.05). When examining the literature on the relationship between caffeine and sleep quality, Bouloukaki et al.’s (2023) [43] study, which investigated sleep quality and fatigue levels among university students during exam periods, included 940 students. This study showed that students’ PSQI scores significantly increased during exam periods, indicating a decline in sleep quality.

Additionally, it was found that caffeine consumption increased during this period and was associated with poor sleep quality. The study by Zunhammer et al. (2014) [44], which examined the relationships between sleep quality and the consumption of legal drugs (alcohol, caffeine, and nicotine) during exam stress among university students, also found that increased caffeine consumption was associated with poor sleep quality. An interesting finding in our study was that individuals who participated in more sports activities were found to smoke more (r = 0.217, *p* < 0.05). Another study by Alotaibi et al. (2020) [45] on university students examined the relationship between sleep quality and mental health. This study found that students who smoked had lower sleep quality and higher levels of depression and anxiety. These findings emphasize that smoking not only negatively affects sleep quality but also has adverse effects on mental health [46,47,48]. The current data show that university students’ PSQI values change periodically and smoking and coffee consumption negatively affect sleep quality.

An unwanted effect that influences the quality of sleep is the appearance of bruxism. At the level of university students, studies show that the prevalence of nocturnal bruxism is increasing due to the stress of academic demands [49]. In a study by Huañec-Paucar et al. (2021) [50], a positive association was found between the level of academic performance and the appearance of bruxism in sleep—36.45% in a sample of 203 students, similar to the age of our sample. At the level of physical education students, the study by Stefanelli et al. (2022) found that 33% of a sample of 178 subjects reported waking bruxism in association with anxiety and stress [51]. Practicing physical activities regularly has been associated with an increase in sleep quality and duration [52,53]. Sleep bruxism significantly impacts sleep quality; it is estimated that sleep bruxism affects 21% of the population [54]. Caffeine consumption and smoking affect episodes of bruxism [55,56]. Specifically, caffeine consumption and certain physical exercises influence the reduction in nocturnal bruxism activity, which in turn leads to improved sleep quality.

Future research directions will be able to focus on monitoring different postures in relation to sleep over certain time units, taking into account other body segments, aiming for an awareness of vicious positives; the impact of sleep characteristics in relation to the specifics of the practiced sport or lifestyle; applying the study to different categories of subjects of different ages and practitioners of different sports, etc. An additional approach to the present cross-sectional study could be the extension to a longitudinal study, by implementing physical activity programs and monitoring the quality of sleep among university students; carrying out a counseling program regarding the effects of caffeine consumption and smoking, as well as the reduction in unwanted effects in correlation with the quality of sleep. Another direction of research may consist in identifying the position of the head–neck relationship by using specific devices to correct certain postural defects.

The practical implications of the present study are aimed at improving posture during sleep by implementing corrective and compensatory exercise programs in daily activities; extending the monitoring of sleep quality and posture to other categories of subjects in correlation with certain daily habits, such as body posture during study periods and during the use of devices and their daily duration; monitoring and optimizing the students’ physical activity and recreation in order to improve the quality of sleep and the posture while during it.

The present study presents several limitations, among which we mention the following: the relatively small number of subjects included in the study; the lack of an interventional program and tracking the results over different periods of time; extending the study to other age categories. Another limitation of the present study concerns the cross-sectional study typology that provides a snapshot of the relationships between sleep quality and posture, without providing evidence of intervention programs; the study data were self-reported by the subjects, and no experimental monitoring was used in specialized laboratories.

## 5. Conclusions

In conclusion, the findings of the study significantly highlight the relationships among caffeine consumption, physical activity levels, and sleep quality. A positive relationship was observed between caffeine consumption and sleep quality, with participants consuming six or more units of caffeine per day exhibiting significantly poorer sleep quality compared to other groups. This result suggests that caffeine consumption may negatively affect sleep quality. On the other hand, a negative relationship was found between physical activity and sleep quality; participants engaging in exercise 3–5 days a week had higher sleep quality scores compared to those who did not participate in physical activity. This finding supports the notion that regular physical activity has a beneficial effect on sleep quality. Additionally, individuals exercising 7 days a week had significantly lower REEDCO scores compared to other groups, indicating that continuous and intense exercise may have certain physical and psychological effects. Pearson correlation analysis revealed positive relationships between age and caffeine consumption, as well as between the frequency of exercise and smoking. Furthermore, increased physical activity was observed to be associated with lower REEDCO scores and improved sleep quality. The relationships among REEDCO, sleep quality, and the head and neck regions also underscore the health impacts of physical activities performed in these areas.

Overall, these findings suggest that reducing caffeine consumption and encouraging regular physical activity can have positive effects on sleep quality and beneficial outcomes for overall health. Further research could provide more detailed insights into the mechanisms underlying these relationships and contribute to the development of strategies aimed at improving sleep quality; future studies could incorporate objective sleep measures (e.g., polysomnography or actigraphy) to validate the findings.

## Figures and Tables

**Table 1:** The relationship between caffeine consumption and poor sleep quality, one-way ANOVA analysis.

Caffeine	X	SD	N	Sum of Squares	df	Mean Square	F	*p*	η^2^	Fark
1 per day	6.135	3.630	52	158.182	3	52.727	4.157	0.008	0.119	4 > 3 4 > 1
2–3 per day	7.500	3.784	34							
4–5 per day	7.000	1.195	8							
6 and above	14.500	3.536	2							

X—arithmetic means; SD—standard deviation; N—number of subjects; η^2^—Eta-squared; *p*—probability; F—value is the ratio between group variation and within-group variation.

**Table 2:** The relationship between participation in sports and sleep quality, one-way ANOVA analysis.

Participation in Sports	X	SD	N	Sum of Squares	df	Mean Square	F	*p*	η^2^	Fark
no	9.105	4.345	19	133.606	3	44.535	3.438	0.020	0.101	4 > 2
1–3 days a week	6.839	3.226	31							
3–5 days a week	5.909	3.844	33							
7 days a week	6.077	2.362	13							

X—arithmetic means; SD—standard deviation; N—number of subjects; η^2^—Eta-squared; *p*—probability; F—value is the ratio between group variation and within-group variation.

**Table 3:** REEDCO scores by level of sports participation, one-way ANOVA analysis.

Participation in Sports	X	SD	N	Sum of Squares	df	Mean Square	F	*p*	η^2^	Fark
no	78.842	5.252	19	1387.325	3	462.442	7.698	<0.001	0.201	4 > 3 2 > 3 1 > 3
1–3 days a week	76.742	8.343	31							
3–5 days a week	73.697	6.498	33							
7 days a week	66.385	11.515	13							

X—arithmetic means; SD—standard deviation; N—number of subjects; η^2^—Eta-squared; *p*—probability; F—value is the ratio between group variation and within-group variation).

**Table 4:** Pearson correlation analysis of parameters affecting sleep quality on PSQI.

Correlations									
	Age	Caffeine	Smoking	Participation in Sports	BMI	PSQI	REEDCO	Right Head	Left Head
Age	1								
Caffeine	0.269 **	1							
Smoking	−0.241 *	−0.187	1						
Participation in Sports	0.079	0.01	0.217 *	1					
BMI	−0.068	−0.027	−0.009	0.089	1				
PSQI	0.083	0.267 **	−0.129	−0.278 **	−0.053	1			
REEDCO	0.004	0.106	−0.101	−0.423 **	−0.102	0.183	1		
Right Head	0.092	0.06	0.067	0.210 *	0.049	−0.023	−0.296 **	1	
Left Head	0.048	−0.016	0.043	0.247 *	0.087	−0.135	−0.463 **	0.392 **	1

BMI—body mass index; ** Correlation is significant at the 0.05 level; * Correlation is significant at the 0.01 level.

## Data Availability

The original contributions presented in the study are included in the article, further inquiries can be directed to the corresponding author.

## References

[1] Orr W.C., Fass R., Sundaram S.S., Scheimann A.O. (2020). The effect of sleep on gastrointestinal functioning in common digestive diseases. Lancet Gastroenterol. Hepatol..

[2] Yang F.N., Xie W., Wang Z. (2022). Effects of sleep duration on neurocognitive development in early adolescents in the USA: A propensity score matched, longitudinal, observational study. Lancet Child Adolesc. Health.

[3] Keshavarz Akhlaghi A.A., Ghalebandi M.F. (2009). Sleep Quality and Its Correlation with General Health in Pre-university Students of Karaj. Iran Iran. J. Psychiatry Behav. Sci. (IJPBS).

[4] Cizza G., Skarulis M., Mignot E. (2005). A link between short sleep and obesity: Building the evidence for causation. Sleep.

[5] Chokroverty S. (2005). Overview of sleep & sleep disorders. Indian J. Med. Res..

[6] Yaffe K., Falvey C.M., Hoang T. (2014). Connections between sleep and cognition in older adults. Lancet Neurol..

[7] Xu C.Y., Zhu K.T., Ruan X.Y., Zhu X.Y., Zhang Y.S., Tong W.X., Li B. (2023). Effect of physical exercise on sleep quality in college students: Mediating role of smartphone use. PLoS ONE.

[8] Kline C.E. (2014). The bidirectional relationship between exercise and sleep: Implications for exercise adherence and sleep improvement. Am. J. Lifestyle Med..

[9] Guembri M.A., Racil G., Tounsi M., Aouichaoui C., Russo L., Migliaccio G.M., Trabelsi Y., Souissi N., Padulo J. (2024). Effects of Ramadan Fasting on Sleep and Physical Fitness among Young Female Handball Players. Children.

[10] Varma P., Postnova S., Knock S., Howard M.E., Aidman E., Rajaratnam S.W.M., Sletten T.L. (2024). SleepSync: Early Testing of a Personalised Sleep–Wake Management Smartphone Application for Improving Sleep and Cognitive Fitness in Defence Shift Workers. Clocks Sleep.

[11] Reid K.J., Baron K.G., Lu B., Naylor E., Wolfe L., Zee P.C. (2010). Aerobic exercise improves self-reported sleep and quality of life in older adults with insomnia. Sleep Med..

[12] Lund H.G., Reider B.D., Whiting A.B., Prichard J.R. (2010). Sleep patterns and predictors of disturbed sleep in a large population of college students. J. Adolesc. Health..

[13] Chandler L., Patel C., Lovecka L., Gardani M., Walasek L., Ellis J., Meyer C., Johnson S., Tang N.K.Y. (2022). Improving university students’ mental health using multi-component and single-component sleep interventions: A systematic review and meta-analysis. Sleep Med..

[14] İyigün G., Angın E., Kırmızıgil B., Öksüz S., Özdil A., Malkoç M. (2017). Üniversite öğrencilerinde uyku kalitesinin mental sağlık, fiziksel sağlık ve yaşam kalitesi ile ilişkisi. J. Exerc. Ther. Rehabil..

[15] Yin J., Jin X., Shan Z., Li S., Huang H., Li P., Peng X., Peng Z., Yu K., Bao W. (2017). Relationship of sleep duration with all-cause mortality and cardiovascular events: A systematic review and dose-response meta-analysis of prospective cohort studies. J. Am. Heart. Assoc..

[16] Buysse D.J. (2014). Sleep health: Can we define it? Does it matter?. Sleep.

[17] Shochat T., Cohen-Zion M., Tzischinsky O. (2014). Functional consequences of inadequate sleep in adolescents: A systematic review. Sleep Med. Rev..

[18] Oksenberg A., Silverberg D.S. (1998). The effect of body posture on sleep-related breathing disorders: Facts and therapeutic implications. Sleep Med. Rev..

[19] Saygın M., Öztürk Ö., Gonca T., Has M., Hayri U.B., Kurt Y., Yağlı M.A., Çalışkan S., Akkaya A., Öztürk M. (2016). Investigation of Sleep Quality and Sleep Disorders in Students of Medicine. Turk. Thorac. J..

[20] Moseley G.L., Nicholas M.K., Hodges P.W. (2004). Pain differs from nonpainful attention-demanding or stressful tasks in its effect on postural control patterns of trunk muscles. Exp. Brain Res..

[21] Solakoğlu Ö., Yalçın P., Dinçer G. (2020). The effects of forward head posture on expiratory muscle strength in chronic neck pain patients: A cross-sectional study. Turk. J. Phys. Med. Rehabil..

[22] Koseki T., Kakizaki F., Hayashi S., Nishida N., Itoh M. (2019). Effect of forward head posture on thoracic shape and respiratory function. J. Phys. Ther. Sci..

[23] Gordon C., Chumlea W.C., Roche A.F., Lohman T.G., Roche A.F., Martorell R. (1988). Stature, recumbent length, and weight. An-Thropometric Standardization Reference Manual. Champaign; Human Kinetics Books.

[24] Trovato B., Roggio F., Sortino M., Zanghì M., Petrigna L., Giuffrida R., Musumeci G. (2022). Postural Evaluation in Young Healthy Adults through a Digital and Reproducible Method. J. Funct. Morphol. Kinesiol..

[25] Irfan U., Asif S., Mumtaz M., Jamal S., Khalid F., Fatima K., Nawaz I., Sheikh N., Rafique H., Aslam I. (2024). Prevalence of Poor Body Posture among Physiotherapists Using APECS. J. Health Rehabil. Res..

[26] Hopkins B.B., Vehrs P.R., Fellingham G.W., George J.D., Hager R., Ridge S.T. (2019). Validity and Reliability of Standing Posture Measurements Using a Mobile Application. J. Manip. Physiol. Ther..

[27] Gumuscu B.H., Kisa E.P., Kara Kaya B., Muammer R. (2023). Comparison of three different exercise trainings in patients with chronic neck pain: A randomized controlled study. Korean J. Pain.

[28] O’Neil M.B., Woodard M., Sosa V., Hunter L., Mulrow C.D., Gerety M.B., Tuley M. (1992). Physical therapy assessment and treatment protocol for nursing home residents. Phys. Ther..

[29] Buysse D.J., Reynolds C.F., Monk T.H. (1989). The Pittsburgh Sleep Quality Index: A new instrument for psychiatric practice and research. Psychiatry Res..

[30] Ağargün M.Y., Kara H., Anlar O. (1996). The reliability and validity the Pittsburgh sleep quality index. Turk. J. Psychiatry.

[31] Nielson C.A., Deegan E.G., Hung A.S.L., Nunes A.J. (2010). Potential effects of sleep deprivation on sensorimotor integration during quiet stance inyoung adults. West. Undergrad. Res. J. Health Nat. Sci..

[32] Stemplewski R., Ciążyńska J., Cyma-Wejchenig M., Maciaszek J. (2023). The effect of sleep deprivation on postural stability among physically active young adults. Sci. Rep..

[33] Fabbri M., Martoni M., Esposito M.J., Brighetti G., Natale V. (2006). Postural control after a night without sleep. Neuropsychologia.

[34] Gomez S., Patel M., Berg S., Magnusson M., Johansson R., Fransson P.A. (2008). Effects of proprioceptive vibratory stimulation on body movement at 24 and 36h of sleep deprivation. Clin. Neurophysiol..

[35] Patel M., Gomez S., Berg S., Almbladh P., Lindblad J., Petersen H., Magnusson M., Johansson R., Fransson P.A. (2008). Effects of 24-h and 36-h sleep deprivation on human postural control and adaptation. Exp. Brain Res..

[36] Bougard C., Lepelley M.C., Davenne D. (2011). The influences of time-ofday and sleep deprivation on postural control. Exp. Brain Res..

[37] Avni N., Avni I., Barenboim E., Azaria B., Zadok D., Kohen-Raz R., Morad Y. (2006). Brief posturographic test as an indicator of fatigue. Psychiatry Clin. Neurosci..

[38] Forsman P., Haeggström E., Wallin A., Toppila E., Pyykkö I. (2007). Daytime changes in postural stability and repeatability of posturographic measurements. J. Occup. Environ. Med..

[39] Kohen-Raz R., Himmelfarb M., Tzur S., Kohen-Raz A., Shub Y. (1996). An initial evaluation of work fatigue and circadian changes as assessed by multiplate posturography. Percept. Mot. Ski..

[40] Alhusami M., Jatan N., Dsouza S., Sultan M.A. (2024). Association between physical activity and sleep quality among healthcare students. Front. Sports Act Living.

[41] Merellano-Navarro E., Bustamante-Ara N., Russell-Guzmán J., Lagos-Hernández R., Uribe N., Godoy-Cumillaf A. (2022). Association between Sleep Quality and Physical Activity in Physical Education Students in Chile in the Pandemic Context: A Cross-Sectional Study. Healthcare.

[42] Croitoru H., Ene Voiculescu V., Ene Voiculescu C., Abramiuc A. (2024). The study on the quality of life of pregnant women in the prepartum period. Ann. “Dunarea De Jos” Univ. Galati. Fascicle XV Phys. Educ. Sport Manag..

[43] Bouloukaki I., Tsiligianni I., Stathakis G., Fanaridis M., Koloi A., Bakiri E., Moudatsaki M., Pouladaki E., Schiza S. (2023). Sleep Quality and Fatigue during Exam Periods in University Students: Prevalence and Associated Factors. Healthcare.

[44] Zunhammer M., Eichhammer P., Busch V. (2014). Sleep quality during exam stress: The role of alcohol, caffeine and nicotine. PLoS ONE.

[45] Alotaibi A.D., Alosaimi F.M., Alajlan A.A., Abdulrahman K.A.B. (2020). The relationship between sleep quality, stress, and academic performance among medical students. J. Fam. Community Med..

[46] Croitoru H., Ene Voiculescu V., Abramiuc A., Ene Voiculescu C. (2024). Implementation and periodization of physical exercise in the postpartum period case study. Ann. “Dunarea De Jos” Univ. Galati. Fascicle XV Phys. Educ. Sport Manag..

[47] Grigoriou I., Kotoulas S.-C., Porpodis K., Spyratos D., Papagiouvanni I., Tsantos A., Michailidou A., Mourelatos C., Mouratidou C., Alevroudis I. (2024). The Interactions between Smoking and Sleep. Biomedicines.

[48] Woo D.H., Park M., Jang S.Y., Park S., Jang S.I. (2023). Association between smoking status and subjective quality of sleep in the South Korean population: A cross-sectional study. Sleep Breath..

[49] Vlăduțu D., Popescu S.M., Mercuț R., Ionescu M., Scrieciu M., Glodeanu A.D., Stănuși A., Rîcă A.M., Mercuț V. (2022). Associations between Bruxism, Stress, and Manifestations of Temporomandibular Disorder in Young Students. Int. J. Environ. Res. Public Health.

[50] Huañec-Paucar C., Ayma-León V., Caballero-García S. (2021). Asociación entre bruxismo autorreportado y rendimiento académico de los estudiantes universitarios. J. Oral Res..

[51] Stefanelli M.L., Meléndez G.J., Kreiner F., Marcelo F.R., Luis I., Scarrone P., Daniela L., Bentancort S. (2022). Self-report of awake bruxism and related factors amongst physical education university students. Odontoestomatología.

[52] Xie Y., Liu S., Chen X.J., Yu H.H., Yang Y., Wang W. (2021). Effects of Exercise on Sleep Quality and Insomnia in Adults: A Systematic Review and Meta-Analysis of Randomized Controlled Trials. Front. Psychiatry.

[53] Alnawwar M.A., Alraddadi M.I., Algethmi R.A., Salem G.A., Salem M.A., Alharbi A.A. (2023). The Effect of Physical Activity on Sleep Quality and Sleep Disorder: A Systematic Review. Cureus.

[54] Zieliński G., Pająk A., Wójcicki M. (2024). Global Prevalence of Sleep Bruxism and Awake Bruxism in Pediatric and Adult Populations: A Systematic Review and Meta-Analysis. J. Clin. Med..

[55] Bertazzo-Silveira E., Kruger C.M., Porto De Toledo I., Porporatti A.L., Dick B., Flores-Mir C., De Luca Canto G. (2016). Association between sleep bruxism and alcohol, caffeine, tobacco, and drug abuse: A systematic review. J. Am. Dent. Assoc..

[56] Weronika F., Mieszko W., Dorian N., Rafal P., Lachowicz G., Mazur G., Martynowicz H. (2023). The effect of coffee and black tea consumption on sleep bruxism intensity based on polysomnographic examination. Heliyon.

